# Stability of Gene Regulatory Networks Modeled by Generalized Proportional Caputo Fractional Differential Equations

**DOI:** 10.3390/e24030372

**Published:** 2022-03-05

**Authors:** Ricardo Almeida, Ravi P. Agarwal, Snezhana Hristova, Donal O’Regan

**Affiliations:** 1 Center for Research and Development in Mathematics and Applications, Department of Mathematics, University of Aveiro, 3810-193 Aveiro, Portugal; ricardo.almeida@ua.pt; 2 Department of Mathematics, Texas A&M University-Kingsville, Kingsville, TX 78363, USA; ravi.agarwal@tamuk.edu; 3 Faculty of Mathematics and Informatics, Plovdiv University “P. Hilendarski”, 4000 Plovdiv, Bulgaria; 4 School of Mathematical and Statistical Sciences, National University of Ireland, H91 TK33 Galway, Ireland; donal.oregan@nuigalway.ie

**Keywords:** model of gene regulatory networks, generalized proportional Caputo fractional derivatives, equilibrium, generalized exponential stability, Lyapunov functions

## Abstract

A model of gene regulatory networks with generalized proportional Caputo fractional derivatives is set up, and stability properties are studied. Initially, some properties of absolute value Lyapunov functions and quadratic Lyapunov functions are discussed, and also, their application to fractional order systems and the advantage of quadratic functions are pointed out. The equilibrium of the generalized proportional Caputo fractional model and its generalized exponential stability are defined, and sufficient conditions for the generalized exponential stability and asymptotic stability of the equilibrium are obtained. As a special case, the stability of the equilibrium of the Caputo fractional model is discussed. Several examples are provided to illustrate our theoretical results and the influence of the type of fractional derivative on the stability behavior of the equilibrium.

## 1. Introduction

Gene expression is the process where the hereditary code of a gene is used for synthesizing proteins and producing the structures of the cell. Genes that code for amino acid sequences are named ‘structural genes’. Gene expression processes include two main stages known as ‘Transcription and translations’. Transcription is the creating of messenger RNA (mRNA) by the enzyme RNA polymerase and the processing of the resulting mRNA molecule. A gene regulatory network consists of a number of genes interacting by proteins. Mathematical models of gene regulatory networks are described and studied in several papers (see, for example, [1,2], for fractional order [3,4,5,6], and with delays [7,8]).

Recently, fractional calculus, fractional derivatives, and fractional integrals of various types have been extensively studied and applied in mathematical modeling. The memory property of fractional derivatives makes them well suited in modeling and describing the complex nature of real-world problems, in comparison to local derivatives (see, for example [9,10,11]).

In this paper, a gene regulated model with the generalized proportional Caputo fractional derivative is set up, and the equilibrium is defined. The generalized exponential stability is introduced and studied via the application of Lyapunov functions and their generalized Caputo proportional fractional derivatives. Generalized proportional Caputo fractional derivatives were recently introduced (see [12,13]); this type of derivative is a generalization of the Caputo fractional derivative, and their application provides wider possibilities for modeling adequately the complexity of real-world problems. The stability of fractional order systems with a proportional Caputo fractional derivatives is quite recent (see, for example, [14,15]). In this paper, some properties of absolute values of Lyapunov functions and their fractional derivatives are discussed, and several examples are provided to illustrate the properties. The advantages of the application of the quadratic Lyapunov functions are considered, and sufficient conditions for generalized exponential stability and asymptotic stability are obtained. Several examples are provided to illustrate the theoretical results and the dependence of the fractional derivative on the behavior of the solutions.

## 2. Notes on Fractional Calculus

We recall the definitions needed in this paper, namely fractional integrals and derivatives (cf. [13]):

The generalized proportional fractional integral of a function u:[a,∞)→R is defined by (as long as all integrals are well defined)

$$\begin{array}{cc} & \left({}_{a}I^{q,\rho }u\right)\left(t\right)=\frac{1}{\rho^{\alpha }\Gamma \left(\alpha \right)}\int_{a}^{t}e^{\frac{\rho -1}{\rho }\left(t-s\right)}{\left(t-s\right)}^{q-1}u\left(s\right)\;ds,\;\;\;t\in \left(a,b\right],\;\;q\geq 0,\;\rho \in \left(0,1\right]. \end{array}$$

The generalized Caputo proportional fractional derivative of a function u:[a,∞)→R is defined by (as long as all integrals are well defined)

$$\begin{array}{cc} & \left({}_{a}^{C}D^{q,\rho }u\right)\left(t\right)=\left({}_{a}I^{1-q,\rho }\left(D^{1,\rho }u\right)\right)\left(t\right)\\ & =\frac{1}{\rho^{1-q}\Gamma \left(1-q\right)}\int_{a}^{t}e^{\frac{\rho -1}{\rho }\left(t-s\right)}{\left(t-s\right)}^{-q}\left(D^{\rho }u\right)\left(s\right)\;ds,\;\;\;t\in \left(a,b\right],\;\;q\in \left(0,1\right),\;\rho \in \left(0,1\right], \end{array}$$

where $\left(D^{\rho }u\right)\left(t\right)=\left(1-\rho \right)u\left(t\right)+\rho u^{\prime }\left(t\right)$.

**Remark** **1.** *Note that the generalized proportional Caputo fractional derivative is defined for $u\in C(\left[a,b\right],R^{n})$ via component-wise.*

**Remark** **2.** *If ρ=1, then the generalized Caputo proportional fractional derivative reduces to the classical Caputo fractional derivative of order q∈(0,1):*

$$\left({}_{a}^{C}D^{q,\rho }u\right)\left(t\right)={}_{a}^{C}D^{q}u\left(t\right)=\frac{1}{\Gamma (1-q)}\int_{a}^{t}{\left(t-s\right)}^{-q}u^{\prime }\left(s\right)\;ds.$$

**Definition** **1.** *We say $u\in C^{q,\rho }\left(\left[t_{0},T\right],R^{n}\right)$ if u(·) is differentiable and the generalized proportional Caputo fractional derivative $\;\left({}_{a}^{C}D^{q,\rho }u\right)\left(t\right)$ exists for all $t\in (t_{0},T]$.*

**Lemma** **1.** *Let q,ρ∈(0,1). Then, the generalized proportional fractional derivative of a constant K∈R is*

(1) $$\begin{array}{cc} & \left({}_{a}^{C}D^{q,\rho }K\right)\left(t\right)=\frac{(1-\rho )K}{\rho^{1-q}\Gamma \left(1-q\right)}\int_{a}^{t}e^{\frac{\rho -1}{\rho }\left(t-s\right)}{\left(t-s\right)}^{-q}\;ds\\ & =\frac{K}{\rho^{1-q}\Gamma \left(1-q\right)}\frac{1}{{\left(\rho -1\right)}^{q}}\left[\Gamma \left(1+q,\frac{\rho -1}{\rho }\left(t-a\right)\right)-\Gamma \left(1+q\right)\right],\;\;\;\;for\;\;t>a. \end{array}$$

**Proposition** **1.** *([13], Remark 3.2) The relation*

(2) $$\left({}_{a}^{C}D^{q,\rho }e^{\frac{\rho -1}{\rho }\left(.\right)}\right)\left(t\right)=0,\;\;\;for\;\;t>a$$

*holds.*

We will use the following property of the Mittag–Leffler function with one parameter, defined by $E_{q}\left(z\right)=\sum_{k=0}^{\infty }\frac{z^{k}}{\Gamma (1+kq)}$ with Γ(ξ) the gamma function.

**Proposition** **2.** *([16], Theorem 1.2) For every q∈(0,1), the function $e^{t}/q-E_{q}\left(t^{q}\right)$ is completely monotonic.*

**Corollary** **1.** *[16] If q∈(0,1), then $E_{q}\left(t^{q}\right)<e^{t}/q,\;t\geq 0$.*

## 3. Some Comments on Properties of the Fractional Derivatives of Lyapunov Functions

One of the most applicable Lyapunov functions is the absolute values Lyapunov function. In connection with this, we will give and discuss some results about their fractional derivatives.

In [17], the following result is proved:

**Proposition** **3.** *([17], Lemma 12) If* $u\in C^{1}\left(\left[0,+\infty \right),R\right)$ *is a continuously differentiable function, and the following relation holds almost everywhere:*

$$\;{}_{t_{0}}^{C}D^{q}\left|u\left(t\right)\right|=\left(sign\;u\left(t\right)\right)\;{}_{t_{0}}^{C}D^{q}u\left(t\right),\;0<q<1.$$

This result is applied by many authors to study the stability of various types of Caputo fractional differential equations and models. For example, this equality is applied in the proof of global Mittag–Leffler stability for fractional-order gene regulatory networks in [3], and to study global uniform asymptotical stability for fractional-order gene regulatory networks with delays in [4,18]. Unfortunately, this equality is not satisfied for all continuously differentiable functions, and we will demonstrate this with an example.

**Example** **1.** *Let u(t)=0.5−t,t≥0.*

*For any t∈(0,0.5) we have u(t)>0, $u^{\prime }\left(t\right)=-1$, sign(0.5−t)=1, |u(t)|=0.5−t, ${\left|u\left(t\right)\right|}^{\prime }=-1$, and*

(3) $$\begin{array}{cc} & {}_{0}^{C}D^{q}\left|u\left(t\right)\right|=-\frac{1}{\Gamma (1-0.5)}\int_{0}^{t}{\left(t-s\right)}^{-0.5}\;ds=-\frac{2t^{0.5}}{\Gamma (0.5)}\\ & =\frac{1}{\Gamma (0.5)}\int_{0}^{t}{\left(t-s\right)}^{-0.5}{\left(0.5-s\right)}^{\prime }\;ds={}_{0}^{C}D^{q}u\left(t\right)=\left(sign\;u\left(t\right)\right)\;{}_{0}^{C}D^{q}u\left(t\right). \end{array}$$

*Let t>0.5. Then u(t)<0, sign(0.5−t)=−1, $u^{\prime }\left(t\right)=-1$, |u(t)|=−0.5+t,${\left|u\left(t\right)\right|}^{\prime }=1$, and*

(4) $$\begin{array}{cc} & {}_{0}^{C}D^{q}\left|u\left(t\right)\right|=\frac{1}{\Gamma (0.5)}\int_{0}^{t}{\left(t-s\right)}^{-0.5}{\left(|u\left(s\right)|\right)}^{\prime }\;ds\\ & =\frac{1}{\Gamma (0.5)}\int_{0}^{0.5}{\left(t-s\right)}^{-0.5}{\left(|u\left(s\right)|\right)}^{\prime }\;ds+\frac{1}{\Gamma (0.5)}\int_{0.5}^{t}{\left(t-s\right)}^{-0.5}{\left(|u\left(s\right)|\right)}^{\prime }\;ds\\ & =-\frac{1}{\Gamma (0.5)}\int_{0}^{0.5}{\left(t-s\right)}^{-0.5}ds+\frac{1}{\Gamma (0.5)}\int_{0.5}^{t}{\left(t-s\right)}^{-0.5}ds\\ & =\frac{1}{\Gamma (0.5)}\left(4{\left(t-0.5\right)}^{0.5}-2t^{0.5}\right)\\ & \neq \frac{2t^{0.5}}{\Gamma (0.5)}=\frac{1}{\Gamma (0.5)}\int_{0}^{t}{\left(t-s\right)}^{-0.5}\;ds\\ & =-\frac{1}{\Gamma (0.5)}\int_{0}^{t}{\left(t-s\right)}^{-0.5}{\left(0.5-s\right)}^{\prime }\;ds=-\frac{1}{\Gamma (0.5)}\int_{0}^{t}{\left(t-s\right)}^{-0.5}{\left(u\left(s\right)\right)}^{\prime }\;ds\\ & =\left(sign\;u\left(t\right)\right)\;{}_{0}^{C}D^{q}u\left(t\right). \end{array}$$

*Therefore, for the Caputo fractional derivative, the equality*

$${}_{0}^{C}D^{q}\left|u\left(t\right)\right|=\left(sign\;u\left(t\right)\right)\;{}_{0}^{C}D^{q}u\left(t\right)$$

*is not true for all $t\in [t_{0},T]$ and any function $u\in C^{1}\left(\left[0,\infty \right),R\right)$.*

Note that in the proof of ([5], Theorem 1), the inequality of the type u(0)u(t)≥0 is applied to prove the equality ${}_{0}^{C}D^{q}\left|u\left(t\right)\right|=\left(sign\;u\left(t\right)\right)\;{}_{0}^{C}D^{q}u\left(t\right)$. Unfortunately, this inequality is not true for all functions. We will illustrate this with an example:

**Example** **2.** *Consider the Caputo fractional differential equation*

$${}_{0}^{C}D^{0.5}u\left(t\right)=-2u\left(t\right)-f\left(u\left(t\right)\right),\;t>0$$

*with u(0)=c,c∈R and f(u)=sin(u)+1.5, which is Lipschitz with a constant L=1 and −0.5≤−f(u)=−sin(u)−1.5≤−2.5. This initial value problem is equivalent to the integral equation*

$$u\left(t\right)=c+\frac{1}{\Gamma (0.5)}\int \frac{f(u(s))}{{\left(t-s\right)}^{0.5}}ds.$$

*Use*

$$\frac{1}{\Gamma (0.5)}\int_{0}^{t}{\left(t-s\right)}^{-0.5}\;ds=2t^{0.5}$$

*and obtain the bounds for the solution:*

$$c-t^{0.5}\leq x\left(t\right)\leq c-5t^{0.5}.$$

**Figure 1:**
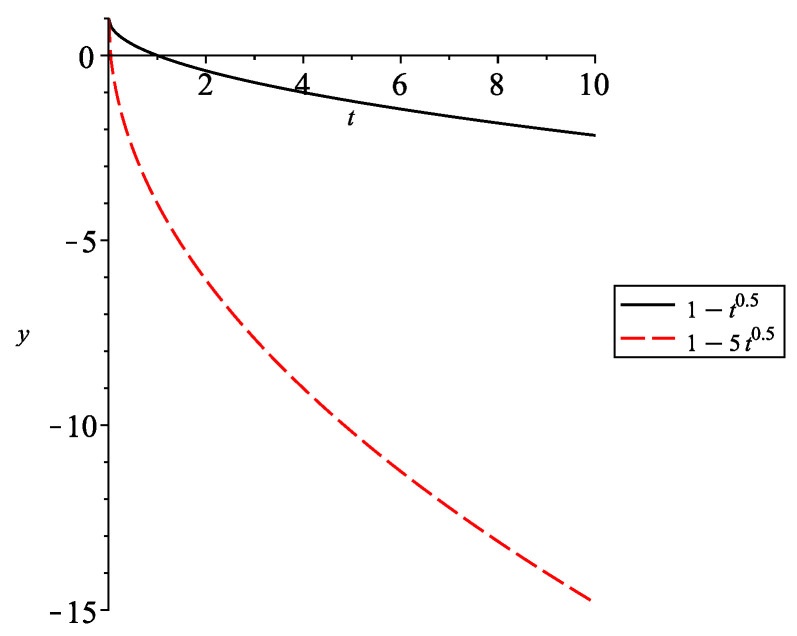
Graph of the bounds of the solution.

*For c=1, we have u(t)<0 for t≥2 (see Figure 1), i.e., u(0)u(t) is not non-negative for all t≥0.*

Note a similar situation occurs when the generalized proportional Caputo fractional derivative is applied. We will illustrate this with an example.

**Example** **3.** *Let u(t)=0.5−t,t≥0, q∈(0,1), and ρ∈(0,1).*

*Case 1.1. Let t∈(0,0.5). Then, we get*

$$(D^{\rho }|u\left(\cdot \right)|)\left(t\right)=0.5-1.5\rho -\left(1-\rho \right)t=\left(sign\;u\left(t\right)\right)\;\left(D^{\rho }u\left(\cdot \right)\right)\left(t\right)=\left(D^{\rho }u\left(\cdot \right)\right)\left(t\right)$$

*and*

(5) $$\begin{array}{cc} & ({}_{0}^{C}D^{q,\rho }|u\left(\cdot \right)|)\left(t\right)=\frac{1}{\rho^{1-q}\Gamma \left(1-q\right)}\int_{0}^{t}e^{\frac{\rho -1}{\rho }\left(t-s\right)}{\left(t-s\right)}^{-q}\left(D^{\rho }u\left(\cdot \right)\right)\left(s\right)\;ds\\ & =\left({}_{0}^{C}D^{q,\rho }u\left(\cdot \right)\right)\left(t\right)=\left(sign\;u\left(t\right)\right)\;\left({}_{0}^{C}D^{q,\rho }u\left(\cdot \right)\right)\left(t\right),\;t\in \left(0,0.5\right). \end{array}$$

*Case 1.2. Let t>0.5. Then, we get*

$$(D^{\rho }|u\left(\cdot \right)|)\left(t\right)=\left(1-\rho \right)\left(t-0.5\right)+\rho =-\left(D^{\rho }u\left(\cdot \right)\right)\left(t\right)=\left(sign\;u\left(t\right)\right)\;\left(D^{\rho }u\left(\cdot \right)\right)\left(t\right)$$

*and*

(6) $$\begin{array}{cc} & ({}_{0}^{C}D^{q,\rho }|u\left(\cdot \right)|)\left(t\right)=\frac{1}{\rho^{1-q}\Gamma \left(1-q\right)}\int_{0}^{t}e^{\frac{\rho -1}{\rho }\left(t-s\right)}{\left(t-s\right)}^{-q}\left(sign\;u\left(s\right)\right)\;\left(D^{\rho }u\left(\cdot \right)\right)\left(s\right)\;ds\\ & =\frac{1}{\rho^{1-q}\Gamma \left(1-q\right)}\int_{0}^{0.5}e^{\frac{\rho -1}{\rho }\left(t-s\right)}{\left(t-s\right)}^{-q}\;\left(D^{\rho }u\left(\cdot \right)\right)\left(s\right)\;ds\\ & \;\;\;\;\;-\frac{1}{\rho^{1-q}\Gamma \left(1-q\right)}\int_{0.5}^{t}e^{\frac{\rho -1}{\rho }\left(t-s\right)}{\left(t-s\right)}^{-q}\left(D^{\rho }u\left(\cdot \right)\right)\left(s\right)\;ds\\ & \neq -\frac{1}{\rho^{1-q}\Gamma \left(1-q\right)}\int_{0}^{t}e^{\frac{\rho -1}{\rho }\left(t-s\right)}{\left(t-s\right)}^{-q}\left(D^{\rho }u\left(\cdot \right)\right)\left(s\right)\;ds\\ & =\left(sign\;u\left(t\right)\right)\;\left({}_{0}^{C}D^{q,\rho }u\left(\cdot \right)\right)\left(t\right). \end{array}$$

*Therefore, for the generalized proportional Caputo fractional derivative, the equality*

$${}_{0}^{C}D^{q,\rho }\left|u\left(t\right)\right|=\left(sign\;u\left(t\right)\right)\;{}_{0}^{C}D^{q,\rho }u\left(t\right)$$

*is not true for all $t\in [t_{0},T]$ and any function $u\in C^{q,\rho }\left(\left[t_{0},T\right],R\right)$.*

We will now prove the correct result. To be general, we will consider the generalized proportional Caputo fractional derivative:

**Lemma** **2.** *Let q∈(0,1), ρ∈(0,1], $u\in C^{q,\rho }\left(\left[t_{0},T\right],R\right)$, and suppose that the sign of u(·) is not changeable in $\left[t_{0},T\right]$. Then, for any $t\in [t_{0},T]$, the equality*

(7) $$\;({}_{t_{0}}^{C}D^{q,\rho }|u\left(\cdot \right)|)\left(t\right)=\left(sign\;u\left(t\right)\right)\;\left({}_{t_{0}}^{C}D^{q,\rho }u\right)\left(t\right)$$

*holds.*

**Proof.**  For any $t\in [t_{0},T]$ we get

$$(D^{\rho }|u\left(\cdot \right)|)\left(t\right)=\left(1-\rho \right)\left(sign\;u\left(t\right)\right)\;u\left(t\right)+\rho \;\left(sign\;u\left(t\right)\right)\;u^{\prime }\left(t\right)=\left(sign\;u\left(t\right)\right)\;\left(D^{\rho }u\left(\cdot \right)\right)\left(t\right)$$

and

$$\begin{array}{cc} & ({}_{t_{0}}^{C}D^{q,\rho }|u\left(\cdot \right)|)\left(t\right)=\frac{1}{\rho^{1-q}\Gamma \left(1-q\right)}\int_{t_{0}}^{t}e^{\frac{\rho -1}{\rho }\left(t-s\right)}{\left(t-s\right)}^{-q}\left(sign\;u\left(s\right)\right)\;\left(D^{\rho }u\left(\cdot \right)\right)\left(s\right)\;ds\\ & =\left(sign\;u\left(t\right)\right)\frac{1}{\rho^{1-q}\Gamma \left(1-q\right)}\int_{t_{0}}^{t}e^{\frac{\rho -1}{\rho }\left(t-s\right)}{\left(t-s\right)}^{-q}\left(D^{\rho }u\left(\cdot \right)\right)\left(s\right)\;ds\\ & =\left(sign\;u\left(t\right)\right)\;\left({}_{0}^{C}D^{q,\rho }u\left(\cdot \right)\right)\left(t\right). \end{array}$$

□

In the case of the Caputo fractional derivative, we obtain the following result:

**Corollary** **2.** *Let $u\in C^{q}\left(\left[t_{0},T\right],R\right)$ and suppose that the sign of u(·) is not changeable in $\left[t_{0},T\right]$. Then, for any $t\in [t_{0},T]$, the equality*

(8) $$\;{}_{t_{0}}^{C}D^{q}\left|u\left(t\right)\right|=\left(sign\;u\left(t\right)\right)\;\left({}_{t_{0}}^{C}D^{q}u\left(t\right)\right)$$

*holds.*

The proof of Corollary 2 follows from Lemma 2 with ρ=1.

**Remark** **3.** *If the function u(·) changes its sign in the interval $\left[t_{0},T\right]$, then because of the memory property of the fractional derivatives (different to integer order derivatives), the equalities (7) and (8) are not true for all points $t\in [t_{0},T]$ (see Example 1).*

When Lyapunov functions are applied to differential equations, including the absolute values Lyapunov function, the type of derivatives of Lyapunov functions is very important and it depends on the type of derivatives in the differential equations.

Let u(·) be a solution of the scalar fractional differential equation $\;{}_{0}^{C}D^{q}u\left(t\right)=f\left(t,u\left(t\right)\right)$. In the literature, several types of fractional derivatives of Lyapunov function V(t,u) are applied. Consider the following derivative, which is called by some authors the upper right-hand derivative in Caputo’s sense or Caputo-type fractional derivative:

$${\;}^{C}D^{q}V\left(u\left(t\right)\right)=\lim_{h\to 0+}\sup\frac{V\left(u\left(t\right)\right)-V(u\left(t\right)-h^{q}f\left(t,u\left(t\right)\right))}{h^{q}}.$$

We will illustrate some of properties of the absolute values Lyapunov function and its derivative given by the above definition with an example.

**Example** **4.** *Let V(x)=|x|. Then, its Caputo-type fractional derivative is*

$$\begin{array}{cc} {\;}^{C}D^{q}\left|u\left(t\right)\right| & =\lim_{h\to 0+}\sup\frac{|u\left(t\right)|-|u\left(t\right)-h^{q}f\left(t,u\left(t\right)\right)|}{h^{q}}=\lim_{\varepsilon \to 0+}\sup\frac{\left|u(t)|-|u(t)-\varepsilon f(t,u(t))\right|}{\varepsilon }\\ & =(sign\left(u\left(t\right)\right)\;f\left(t,u\left(t\right)\right)=(sign\left(u\left(t\right)\right)\;{}_{0}^{C}D^{q}u\left(t\right), \end{array}$$

*where $\varepsilon =h^{q}$.*

*However, the derivative ${\;}^{C}D^{q}V\left(u\left(t\right)\right)$ has no memory and ${\;}^{C}D^{q}V\left(u\left(t\right)\right)\neq \;{}_{0}^{C}D^{q}V\left(u\left(t\right)\right)$, so we could not conclude that ${\;}^{C}D^{q}|u\left(t\right)|=(sign\left(u\left(t\right)\right){\;}^{C}D^{q}u\left(t\right)$.*

**Remark** **4.** *From Example 1, Example 2, and Example 4, it could be seen that in the case of Caputo fractional differential equations, for the Lyapunov function*

$$V\left(t,x\right)=\sum_{k=1}^{n}\left|x_{i}\right|,\;x=\left(x_{1},x_{2},\cdots ,x_{n}\right),$$

*we have:*

- *If its Caputo fractional derivative is applied, then the inequality*
$$\;{}_{0}^{C}D^{q}V\left(t,x\left(t\right)\right)\neq \sum_{i=1}^{n}\left(sign\;x_{i}\left(t\right)\right)\;{}_{0}^{C}D^{q}x_{i}\left(t\right)$$
  *holds in the general case (see Example 1–Case 2). According to Lemma 2, the equality is true only in a particular case;*
- *If its Caputo-type fractional derivative ${\;}^{C}D^{q}\left|u\left(t\right)\right|$ is applied, then in the general case*
$${\;}^{C}D^{q}V\left(u\left(t\right)\right)\neq \sum_{i=1}^{n}\left(sign\;x_{i}\left(t\right)\right)\;{}^{C}D^{q}x_{i}\left(t\right).$$

*Before the application of the equality (8), one needs to prove that the solution has a constant sign (see Corollary 2).*

**Remark** **5.** *The situation mentioned in Remark 4 is true also for generalized proportional Caputo fractional differential equations and the absolute values Lyapunov function.*

According to the above discussions, in this section, the Lyapunov function of the type

$$V\left(t,x\right)=\sum_{k=1}^{n}x_{i}^{2},\;x=\left(x_{1},x_{2},\cdots ,x_{n}\right)$$

is appropriate to apply to fractional differential equations using the following result:

**Lemma** **3.** *[14] Let q∈(0,1),ρ∈(0,1], and $u\in C^{q,\rho }\left(\left[t_{0},\infty \right),R^{n}\right)$. Then, for any $t\geq t_{0}$, the inequality*

$$\;({}_{t_{0}}^{C}D^{q,\rho }\left(u^{T}\left(\cdot \right)u\left(\cdot \right)\right)\left(t\right)\leq 2\;x^{T}\left(t\right)\;\left({}_{t_{0}}^{C}D^{q,\rho }u\right)\left(t\right)$$

*holds.*

**Remark** **6.** *Note that in the special case ρ=1 of Lemma 3, i.e., the application of Caputo fractional derivative, the result is proved in [19,20].*

**Remark** **7.** *Note that quadratic Lyapunov functions for Caputo fractional order time-delayed gene regulatory networks are applied in [7].*

## 4. Statement of the Problem

In this paper, we will consider a class of fractional order gene regulatory networks modeled by a generalized proportional Caputo fractional derivative for 0<q<1, ρ∈(0,1]:

(9) $$\begin{array}{cc} & \;\left({}_{0}^{C}D^{q,\rho }x_{i}\right)\left(t\right)=-d_{i}x_{i}\left(t\right)+\sum_{k=1}^{N}a_{ik}h_{k}\left(y_{k}\left(t\right)\right)+I_{i},\;\;t>0,\;\;i=1,2,\cdots ,N,\\ & \;\left({}_{0}^{C}D^{q,\rho }y_{i}\right)\left(t\right)=-b_{i}y_{i}\left(t\right)+c_{i}x_{i}\left(t\right),\;\;t>0,\;\;i=1,2,\cdots ,N,\\ & x_{i}\left(0\right)=x_{i}^{0},\;\;y_{i}\left(0\right)=y_{i}^{0},\;\;i=1,2,\cdots ,N, \end{array}$$

where $x_{j}^{0},y_{j}^{0}\in R$, $\;x_{j}\left(t\right),y_{j}\left(t\right),\;j=1,2,\cdots ,N,$ denote the concentrations of messenger ribonucleic acid (mRNA) and protein of the *j*-th node at time *t*, respectively, $d_{j}$ and $b_{j}$ are degradation velocities of mRNA and protein, respectively, $c_{j}>0$ is the translation rate, the functions $\;h_{k}\in C\left(R,R\right),\;k=1,2,\cdots ,N,$ represent the activator initiates of protein of mRNA, and the coupling matrix of the network $A=\left(a_{jk}\right)\in R^{N\times N}$ is described by

$$a_{jk}=\left\{\begin{array}{cc} -\gamma_{jk} & k\;\mathrm{is}\;a\;\mathrm{repressor}\;\mathrm{of}\;\mathrm{gene}\;j\;\\ 0 & k\;\mathrm{does}\;\mathrm{not}\;\mathrm{regulate}\;\mathrm{gene}\;j\\ \gamma_{jk} & k\;\mathrm{is}\;a\;\mathrm{initiator}\;\mathrm{of}\;\mathrm{gene}\;j, \end{array}\right.$$

and $I_{j}=\sum_{k\in J}a_{jk}$, where J is the set of all repressors of gene *j*.

**Remark** **8.** *Commonly, the activator functions $h_{k}\left(\cdot \right),\;k=1,2,\cdots ,N,$ are indicated in the Hill form $h_{k}\left(s\right)=\frac{s^{\beta_{k}}}{\alpha_{k}^{\beta_{k}}+s^{\beta_{k}}},\;s\in R$, where $\beta_{k}$ are the Hill coefficients and $\alpha_{k}\geq 0$ are constants.*

**Remark** **9.** *Note that the model (9) is studied in the case of the Caputo fractional derivative and the absolute value Lyapunov function is applied (see Remark 4).*

Introduce the following assumptions:

(A1) The activator functions $h_{k}\left(\cdot \right),\;k=1,2,\cdots ,N$ are increasing and there exist constants $\gamma_{k}>0$ such that for any u,v∈R with u≠v, the inequalities

$$0\leq \frac{h_{k}\left(u\right)-h_{k}\left(v\right)}{u-v}\leq \gamma_{k},\;k=1,2,\cdots ,N$$

hold.

(A2) There exist positive constants $\mu_{k},\mu_{N+k},\;k=1,2,\cdots ,N$ such that the coefficients in (9) satisfy the inequalities

$$\begin{array}{cc} & \sum_{j=1}^{N}\gamma_{j}\left|a_{kj}\right|+\frac{\mu_{N+k}}{\mu_{k}}c_{k}<2d_{k},\;\;\;k=1,2,\cdots ,N,\\ & -2b_{k}c_{k}+\gamma_{k}\sum_{j=1}^{N}\frac{\mu_{j}}{\mu_{N+k}}\left|a_{jk}\right|<2b_{k},\;\;k=1,2,\cdots ,N. \end{array}$$

**Remark** **10.** *From Assumption (A1), it follows that Lemma 2 is applicable to the solutions of (9) and equality (7) holds; i.e., the absolute value Lyapunov function is applicable to (9).*

From Lemma 1, it follows that the generalized proportional Caputo fractional derivative of a nonzero constant is not zero, and applying Corollary 1, we introduce the following definition.

**Definition** **2.** *The couple of functions*

$$\left(x*\left(t\right),y*\left(t\right)\right)=\left(Ce^{\frac{\rho -1}{\rho }t},Qe^{\frac{\rho -1}{\rho }t}\right),$$

*with $C=\left(C_{1},C_{2},\cdots ,C_{N}\right)\in R^{N},\;Q=\left(Q_{1},Q_{2},\cdots ,Q_{N}\right)\in R^{N},\;C_{i},Q_{i}=const$, is called an equilibrium of (9) if*

(10) $$\begin{array}{cc} & d_{j}C_{j}e^{\frac{\rho -1}{\rho }t}=\sum_{k=1}^{N}a_{jk}h_{k}\left(Q_{k}e^{\frac{\rho -1}{\rho }\left(t\right)}\right)+I_{j},\;\;t>t_{0},\;\;i=1,2,\cdots ,N,\\ & \;b_{j}Q_{j}=c_{j}C_{j},\;\;t>t_{0},\;\;i=1,2,\cdots ,N. \end{array}$$

**Remark** **11.** *Note that in the case of Caputo fractional derivative (ρ=1), the defined equilibrium in Definition 2 coincides with the one known in the literature (see, for example, [3]).*

**Definition** **3.** The equilibrium X*(t)=(x*(t),y*(t)) of the model (9) is generalized exponentially stable if there exist constants M,λ>0 such that

$$∥X\left(t\right)-X*\left(t\right)∥\leq M∥X^{0}-X*\left(0\right)∥e^{\frac{\rho -1}{2\rho }t}\sqrt{E_{q}\left(-\lambda {\left(\frac{t}{\rho }\right)}^{q}\right)},\;\;t\geq 0,$$

*where $X^{0}=\left(x^{0},y^{0}\right)$ is the solution of (9) with initial values X(t)=(x(t),y(t)).*

Use the transformations

$$u_{j}\left(t\right)=x_{j}\left(t\right)-C_{j}e^{\frac{\rho -1}{\rho }t},\;v_{j}\left(t\right)=y_{j}\left(t\right)-Q_{j}e^{\frac{\rho -1}{\rho }t}.$$

Then, (9) can be written in the form

(11) $$\begin{array}{cc} & \;\left({}_{0}^{C}D^{q,\rho }u_{i}\right)\left(t\right)=-d_{i}u_{i}\left(t\right)+\sum_{k=1}^{N}a_{ik}H_{k}\left(t,v_{k}\left(t\right)\right),\;\;t>t_{0},\;\;i=1,2,\cdots ,N,\\ & \;\left({}_{0}^{C}D^{q,\rho }v_{j}\right)\left(t\right)=-b_{j}v_{j}\left(t\right)+c_{j}u_{j}\left(t\right),\;\;t>t_{0},\;\;j=1,2,\cdots ,N,\\ & u_{j}\left(0\right)=x_{j}^{0}-C_{j},\;\;v_{j}\left(0\right)=y_{j}^{0}-Q_{j},\;\;j=1,2,\cdots ,N, \end{array}$$

where

$$H_{j}\left(t,v_{k}\right)=h_{k}\left(v_{k}+Q_{k}e^{\frac{\rho -1}{\rho }t}\right)-h_{k}\left(Q_{k}e^{\frac{\rho -1}{\rho }t}\right).$$

The system (11) has a zero equilibrium.

The goal of our paper is to study the exponential and asymptotic stability of the equilibrium of (9); equivalently, we also study the stability properties of the zero solution of the IVP for FrDE (11).

We will apply quadratic Lyapunov functions, and in connection with this, we will use the following result:

**Lemma** **4.** *([14], Lemma 2) Let the function $U\left(t\right)=\left(u\left(t\right),v\left(t\right)\right)\in C^{q,\rho }\left(\left[0,\infty \right),R^{2N}\right)$, with $u\left(\cdot \right)\in C^{q,\rho }\left(\left[0,\infty \right),R^{N}\right),\;v\left(\cdot \right)\in C^{q,\rho }\left(\left[0,\infty \right),R^{N}\right)$ be a solution of (11), and suppose that, for any t≥0, the inequality*

(12) $$\;{}_{0}^{C}D^{q,\rho }{∥U\left(t\right)∥}^{2}\leq 0$$

*holds. Then,*

(13) $$∥U\left(t\right)∥\leq ∥U^{0}∥e^{\frac{\rho -1}{2\rho }t},\;\;\;\;\;forall\;t\geq 0,$$

*where*

$$U^{0}=\left(x_{1}^{0}-C_{1},x_{2}^{0}-C_{2},\cdots ,x_{N}^{0}-C_{N},y_{1}^{0}-Q_{1},\cdots ,y_{N}^{0}-Q_{N}\right).$$

**Lemma** **5.** *([14], Lemma 3) Let the function $U\left(t\right)=\left(u\left(t\right),v\left(t\right)\right)\in C^{q,\rho }\left(\left[0,\infty \right),R^{2N}\right)$, with $u\left(\cdot \right)\in C^{q,\rho }\left(\left[0,\infty \right),R^{N}\right),v\left(\cdot \right)\in C^{q,\rho }\left(\left[0,\infty \right),R^{N}\right)$, be a solution of (11) and suppose that, for any t≥0, the inequality*

(14) $$\;{}_{0}^{C}D^{q,\rho }{∥U\left(t\right)∥}^{2}\leq -K{∥U\left(t\right)∥}^{2}$$

*holds, where K>0 is a constant. Then,*

(15) $$∥U\left(t\right)∥\leq ∥U^{0}∥e^{\frac{\rho -1}{2\rho }t}\sqrt{E_{q}\left(-K{\left(\frac{t}{\rho }\right)}^{q}\right)},\;\;\;\;\;forall\;t\geq 0,$$

*where*

$$U^{0}=\left(x_{1}^{0}-C_{1},x_{2}^{0}-C_{2},\cdots ,x_{N}^{0}-C_{N},y_{1}^{0}-Q_{1},\cdots ,y_{N}^{0}-Q_{N}\right).$$

**Theorem** **1.** Let the assumptions (A1) and (A2) be satisfied, and assume that there exists an equilibrium X*(t)=(x*(t),y*(t)) of the model (9). Then, the equilibrium of the model (9) is generalized exponentially stable.

**Proof.**  The generalized exponentially stability of equilibrium of the model (9) is equivalent to the generalized exponential stability of the zero solution of (11).

Consider the Lyapunov function

$$V\left(U\right)={∥MU∥}^{2}=\sum_{k=1}^{N}\mu_{k}u_{k}^{2}+\sum_{k=1}^{N}\mu_{N+k}v_{k}^{2},$$

where

$$M=(\sqrt{\mu_{1}},\sqrt{\mu_{2}},\cdots ,\sqrt{\mu_{N}},\sqrt{\mu_{N+1}},\cdots ,\sqrt{\mu_{2N}}).$$

Let U(·) be a solution of (11). According to Lemma 3, we obtain

(16) $$\begin{array}{cc} & \;{}_{0}^{C}D^{q,\rho }V\left(U\left(t\right)\right)=\sum_{k=1}^{N}\mu_{k}\;{}_{0}^{C}D^{q,\rho }u_{k}^{2}\left(t\right)+\sum_{k=1}^{N}\mu_{N+k}\;{}_{0}^{C}D^{q,\rho }v_{k}^{2}\left(t\right)\\ & \leq 2\sum_{k=1}^{N}u_{k}\left(t\right)\;{}_{0}^{C}D^{q,\rho }u_{k}\left(t\right)+2\sum_{k=1}^{N}v_{k}\left(t\right)\;{}_{0}^{C}D^{q,\rho }v_{k}\left(t\right)\\ & =-2\sum_{k=1}^{N}\mu_{k}d_{k}u_{k}^{2}\left(t\right)+2\sum_{k=1}^{N}\sum_{j=1}^{N}\mu_{k}a_{kj}u_{k}\left(t\right)H_{j}\left(t,v_{j}\left(t\right)\right)\\ & \;\;\;-2\sum_{k=1}^{N}\mu_{N+k}b_{k}v_{k}^{2}\left(t\right)+2\sum_{k=1}^{N}\mu_{N+k}c_{k}v_{k}\left(t\right)u_{k}\left(t\right). \end{array}$$

From assumption (A2), it follows that

$$\frac{H_{k}\left(t,v_{k}\left(t\right)\right)}{v_{k}\left(t\right)}=\frac{h_{k}\left(v_{k}\left(t\right)+Q_{k}e^{\frac{\rho -1}{\rho }t}\right)-h_{k}\left(Q_{k}e^{\frac{\rho -1}{\rho }t}\right)}{v_{k}\left(t\right)}\leq \gamma_{k},\;k=1,2,\cdots ,N,$$

and thus

(17) $$\begin{array}{cc} & \;{}_{0}^{C}D^{q,\rho }V\left(U\left(t\right)\right)\leq -2\sum_{k=1}^{N}\mu_{k}d_{k}u_{k}^{2}\left(t\right)+2\sum_{k=1}^{N}\sum_{j=1}^{N}\mu_{k}|a_{kj}\left|\;\right|u_{k}\left(t\right)|\gamma_{j}\left|v_{j}\left(t\right)\right|\\ & \;\;\;-2\sum_{k=1}^{N}\mu_{N+k}b_{k}v_{k}^{2}\left(t\right)+2\sum_{k=1}^{N}\mu_{N+k}c_{k}|v_{k}\left(t\right)|\;|u_{k}\left(t\right)|\\ & \leq -2\sum_{k=1}^{N}\mu_{k}d_{k}u_{k}^{2}\left(t\right)+\sum_{k=1}^{N}\mu_{k}\sum_{j=1}^{N}|a_{kj}|\gamma_{j}u_{k}^{2}\left(t\right)+\sum_{k=1}^{N}\sum_{j=1}^{N}\mu_{k}\left|a_{kj}\right|\gamma_{j}v_{j}^{2}\left(t\right)\\ & \;\;\;-2\sum_{k=1}^{N}\mu_{N+k}b_{k}v_{k}^{2}\left(t\right)+\sum_{k=1}^{N}\mu_{N+k}c_{k}u_{k}^{2}\left(t\right)+\sum_{k=1}^{N}\mu_{N+k}c_{k}v_{k}^{2}\left(t\right)\\ & \leq \sum_{k=1}^{N}\left[-2d_{k}+\sum_{j=1}^{N}\gamma_{j}\left|a_{kj}\right|+\frac{\mu_{N+k}}{\mu_{k}}c_{k}\right]\mu_{k}u_{k}^{2}\left(t\right)\\ & \;\;\;\sum_{k=1}^{N}\left[-2b_{k}+c_{k}+\gamma_{k}\sum_{j=1}^{N}\frac{\mu_{j}}{\mu_{N+k}}\left|a_{jk}\right|\right]\mu_{N+k}v_{k}^{2}\left(t\right)\\ & \leq -\lambda V(U(t)), \end{array}$$

where

$$\lambda =\max_{k=1,2,\cdots ,N}\left\{2d_{k}-\sum_{j=1}^{N}\gamma_{j}|a_{kj}|-\frac{\mu_{N+k}}{\mu_{k}}c_{k},2b_{k}-c_{k}-\gamma_{k}\sum_{j=1}^{N}\frac{\mu_{j}}{\mu_{N+k}}\left|a_{jk}\right|\right\}.$$

According to Lemma 5, the inequality

(18) $$\begin{array}{cc} & \mu_{min}∥U\left(t\right)∥=\mu_{min}\sqrt{\sum_{k=1}^{N}u_{k}^{2}\left(t\right)+\sum_{k=1}^{N}v_{k}^{2}\left(t\right)}\leq \sqrt{\sum_{k=1}^{N}\mu_{k}u_{k}^{2}\left(t\right)+\sum_{k=1}^{N}\mu_{N+k}u_{k}^{2}\left(t\right)}\\ & \;\;\;\;=∥MU\left(t\right)∥\leq ∥MU^{0}∥e^{\frac{\rho -1}{2\rho }t}\sqrt{E_{q}\left(-\lambda {\left(\frac{t}{\rho }\right)}^{q}\right)}\leq \mu_{max}∥U^{0}∥e^{\frac{\rho -1}{2\rho }t}\sqrt{E_{q}\left(-\lambda {\left(\frac{t}{\rho }\right)}^{q}\right)} \end{array}$$

holds, where

$$\mu_{min}=\min\left\{\min_{k=1,2,\cdots ,N}\sqrt{\mu_{k}},\min_{k=1,2,\cdots ,N}\sqrt{\mu_{N+k}}\right\}$$

and

$$\mu_{max}=\max\left\{\max_{k=1,2,\cdots ,N}\sqrt{\mu_{k}},\max_{k=1,2,\cdots ,N}\sqrt{\mu_{N+k}}\right\},$$

or

$$∥U\left(t\right)∥\leq \frac{\mu_{max}}{\mu_{min}}∥U^{0}∥e^{\frac{\rho -1}{2\rho }t}\sqrt{E_{q}\left(-\lambda {\left(\frac{t}{\rho }\right)}^{q}\right)}.$$

□

**Corollary** **3.** *Let the conditions of Theorem 1 be satisfied. Then, the equilibrium of the model (9) is asymptotically stable, i.e.,*

$$\lim_{t\to \infty }∥x\left(t\right)-x*\left(t\right)∥=0\;and\;\lim_{t\to \infty }∥y\left(t\right)-y*\left(t\right)∥=0.$$

## 5. Applications

**Application 1**

We will consider the model of three repressor-protein concentrations, $p_{i}$, and their corresponding mRNA concentrations, $m_{i},\;i=1,2,3$, which are defined and studied in [21] when the kinetics of the system are determined by ordinary differential equations. To have a more appropriate model, we will adopt this model and use generalized proportional Caputo fractional derivatives; i.e., we will consider the model

(19) $$\begin{array}{cc} & \;\left({}_{0}^{C}D^{q,\rho }m_{i}\right)\left(t\right)=-m_{i}+\frac{\alpha }{\left(1+p_{i}^{n}\right)}+\alpha_{0}\\ & \;\left({}_{0}^{C}D^{q,\rho }p_{i}\right)\left(t\right)=-\beta \left(p_{i}-m_{i}\right),\;\;t>0,\;i=1,2,3, \end{array}$$

where (see [21]):

- The number of protein copies per cell produced from a given promoter type during continuous growth is $\alpha_{0}$ in the presence of saturating amounts of repressor and $\alpha +\alpha_{0}$ in its absence;
- β is the ratio of the protein decay rate to the mRNA decay rate;
- *n* is a Hill coefficient.

System (19) is similar to (9) with $d_{i}=1,\;b_{i}=c_{i}=\beta$, $h_{k}\left(u\right)=\frac{1}{1+u^{n}}$, $a_{ii}=\alpha$ and $a_{ik}=0$ for k≠i, $I_{i}=\alpha_{0}$.

Take n=2,α=1.3, and β=1. Thus, $\gamma_{k}=0.649519$, $m_{i}=m_{3+i}=1$, and

$$-2b_{k}+c_{k}+\gamma_{k}\sum_{j=1}^{N}\frac{\mu_{j}}{\mu_{N+k}}\left|a_{jk}\right|=-\beta +\left(0.649519\right)\cdot \left(1.3\right)\frac{\mu_{i}}{\mu_{3+i}}=-1+0.844375=-0.155625<0$$

and

$$-2d_{k}+\sum_{j=1}^{N}\gamma_{j}\left|a_{kj}\right|+\frac{\mu_{N+k}}{\mu_{k}}c_{k}=-2+\left(0.649519\right)\cdot \left(1.3\right)+1=-0.155625<0,$$

i.e., assumptions (A1) and (A2) are satisfied. According to Theorem 1, if there exists an equilibrium $E*=\left(\tilde{m_{1}},\tilde{m_{2}},\tilde{m_{3}},\tilde{p_{1}},\tilde{p_{2}},\tilde{p_{3}}\right))$ of (19), then it is generalized exponential stable.

*Case 1*. *Caputo fractional derivative*, i.e., ρ=1. The equilibrium $\tilde{m_{i}},\tilde{p_{i}},i=1,2,3$ is a solution of the system

(20) $$\tilde{m_{i}}=\tilde{p_{i}},\;\tilde{m_{i}}=\frac{\alpha }{\left(1+{\tilde{p_{i}}}^{n}\right)}+\alpha_{0}.$$

The system (20) has a solution for every value of α and $\alpha_{0}$.

Consider a particular case of α=1.3 and $\alpha_{0}=0$. Then, the equilibrium is $\tilde{m_{i}}=\tilde{p_{i}}=0.795876,\;i=1,2,3$.

**Figure 2:**
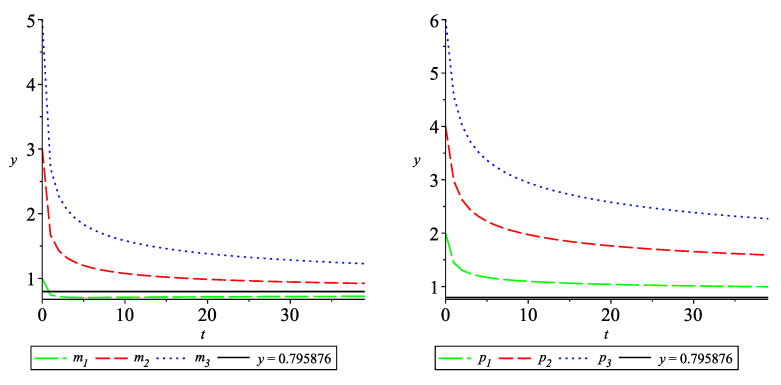
Convergence of the solutions of (19) with q=0.3,ρ=1 to the equilibrium 0.795876.

The solutions $m_{i}\left(\cdot \right),\;i=1,2,3$ are given in Figure 2 (left), and the solutions $p_{i}\left(\cdot \right),\;$i=1,2,3, are given in Figure 2 (right). It could be seen that all components of the solution approach the equilibrium 0.795876.

Note that problem (19) is considered in [3] with $\alpha =2.5,\;\alpha_{0}=0$. However, in this case, the equilibrium is $\tilde{m_{i}}=\tilde{p_{i}}=1.11475$, which does not correspond to the provided graphs.

Let $\alpha =1.3,\;\alpha_{0}=2$. Then, the equilibrium is $\tilde{m_{i}}=\tilde{p_{i}}=2.21938,\;i=1,2,3$.

**Figure 3:**
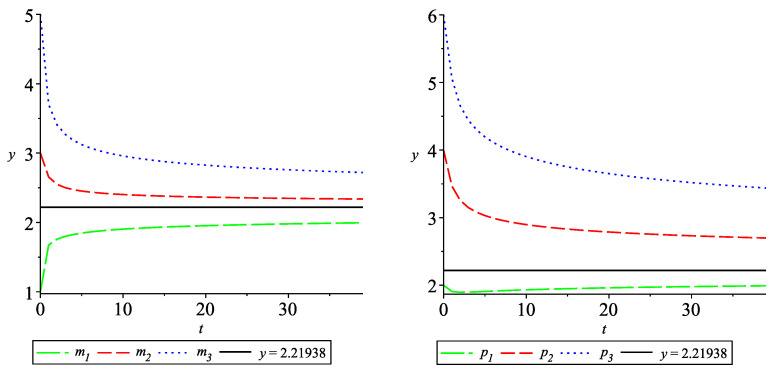
Convergence of the solution of (19) with q=0.3,ρ=1 to the equilibrium 2.21938.

The solutions $m_{i}\left(\cdot \right),\;i=1,2,3$ are given in Figure 3 (left) and the solutions $p_{i}\left(\cdot \right),\;$i=1,2,3, are given in Figure 3 (right). It could be seen that all components of the solution approach 2.21938.

*Case 2*. *Generalized proportional Caputo fractional derivative*, i.e., ρ∈(0,1).

Since $\;{}_{0}^{C}D^{q,\rho }0\neq 0$ and $\;{}_{0}^{C}D^{q,\rho }e^{\frac{\rho -1}{\rho }t}=0,\;t\geq 0$, the equilibrium $\tilde{m_{i}}=\tilde{p_{i}}=C_{j}e^{\frac{\rho -1}{\rho }t},$i=1,2,3, is a solution of the system

(21) $$C_{j}e^{\frac{\rho -1}{\rho }t}=\frac{\alpha }{1+C_{j}^{2}e^{2\frac{\rho -1}{\rho }t}}+\alpha_{0},\;\;t>0.$$

**Figure 4:**
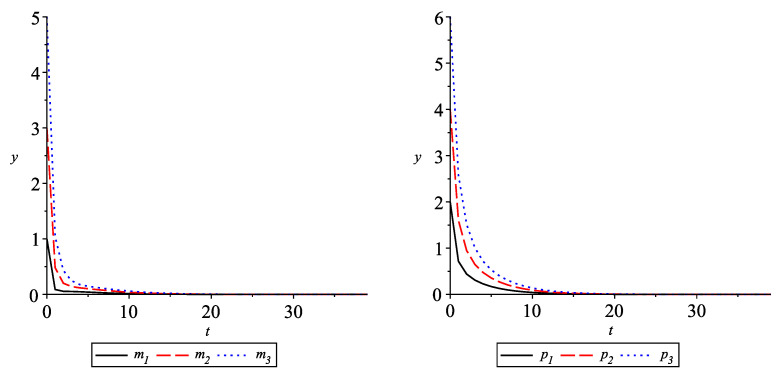
Solution of system (19) with q=0.3,ρ=0.8.

*Case 2.1*. Let $\alpha_{0}=-1.3$. Then, the system (21) has zero solution w.r.t. $C_{j}$ and the system (19) have a zero equilibrium. The solutions $m_{i}\left(\cdot \right),\;i=1,2,3$ are given in Figure 4 (left) and the solutions $p_{i}\left(\cdot \right),\;i=1,2,3$ are given in Figure 4 (right). It could be seen that all components approach the equilibrium 0.

*Case 2.2*. Let $\alpha_{0}\neq -1.3$. Then, the system (21) has no solution w.r.t. $C_{j},j=1,2,3$, and the system (19) has no equilibrium, and we could not apply Theorem 1.

**Application 2**

**Figure 5:**
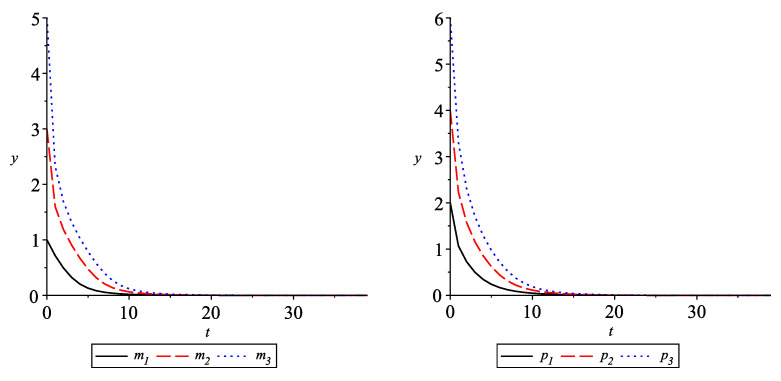
Solution of system (22) with q=0.3,ρ=0.8.

Consider the model of three repressor-protein concentrations, $p_{i}$, and their corresponding mRNA concentrations, $m_{i},\;i=1,2,3$, (19) with the activator functions $h_{k}\left(s\right)=\frac{s^{\beta_{k}}}{\alpha_{k}^{\beta_{k}}+s^{\beta_{k}}},\;s\in R$; i.e., consider

(22) $$\begin{array}{cc} & \;\left({}_{0}^{C}D^{q,\rho }m_{i}\right)\left(t\right)=-m_{i}+\frac{\alpha p_{i}^{n}}{\left(1+p_{i}^{n}\right)}+\alpha_{0},\\ & \;\left({}_{0}^{C}D^{q,\rho }p_{i}\right)\left(t\right)=-\beta \left(p_{i}-m_{i}\right),\;i=1,2,3,\;\;\;t>0, \end{array}$$

with $a_{0}=0$. The system (8) has a zero equilibrium. Take n=2,α=1.3, and β=1. Thus, $\gamma_{k}=0.649519$, $m_{k}=m_{3+k}=1,\;k=1,2,3,$ and assumptions (A1) and (A2) are satisfied. According to Theorem 1, the zero equilibrium is generalized exponential stable. The graphs of the solutions $m_{i}\left(\cdot \right)$ and $p_{i}\left(\cdot \right),\;i=1,2,3$, of system (22) are given in Figure 5 (left) and Figure 5 (right), respectively, with n=2, ρ=0.8,q=0.3, α=1.3, $\alpha_{0}=0$, β=1, with initial values $m_{1}\left(0\right)=1,\;m_{2}\left(0\right)=3,\;m_{3}\left(0\right)=5,\;p_{1}\left(0\right)=2,\;p_{2}\left(0\right)=4,\;p_{3}\left(0\right)=6$.

**Application 3**

Consider the general model describing the dynamics of the interacting defects in the genome and in the proteome with the generalized proportional fractional derivative:

(23) $$\begin{array}{cc} & \;\left({}_{0}^{C}D^{q,\rho }u\right)\left(t\right)=pv\left(t\right)-\alpha u\left(t\right)+f\left(t\right),\\ & \;\left({}_{0}^{C}D^{q,\rho }v\right)\left(t\right)=\beta GKu\left(t\right)-\delta v\left(t\right)+Gg\left(t\right),\;\;t>0, \end{array}$$

where β is the coupling rate constant characterizing the regulation of gene expression by the proteins, *K* is the average number of genes regulated by any single protein and represents a simple measure of the overall connectivity of the genetic network, *c* reflects the combined efficiency of proteolysis and heat shock response systems, mediating the degradation and refolding of misfolded proteins, respectively, whereas δ characterizes the DNA repair rate, the “force” terms, f(·) and g(·) characterize the proteome and genome damage rates, respectively, and *G* is the total genome size.

Let $f\left(t\right)=Ae^{\frac{\rho -1}{\rho }t}$ and $g\left(t\right)=Be^{\frac{\rho -1}{\rho }t}$. Then, the model (23) has equilibrium

$$\left(\tilde{u},\tilde{v}\right)=\left(\frac{GB\alpha +A\beta GK}{\alpha \delta -\beta GKp}e^{\frac{\rho -1}{\rho }t},\frac{A\delta +GBp}{\alpha \delta -\beta GKp}e^{\frac{\rho -1}{\rho }t}\right),\;t>0.$$

**Figure 6:**
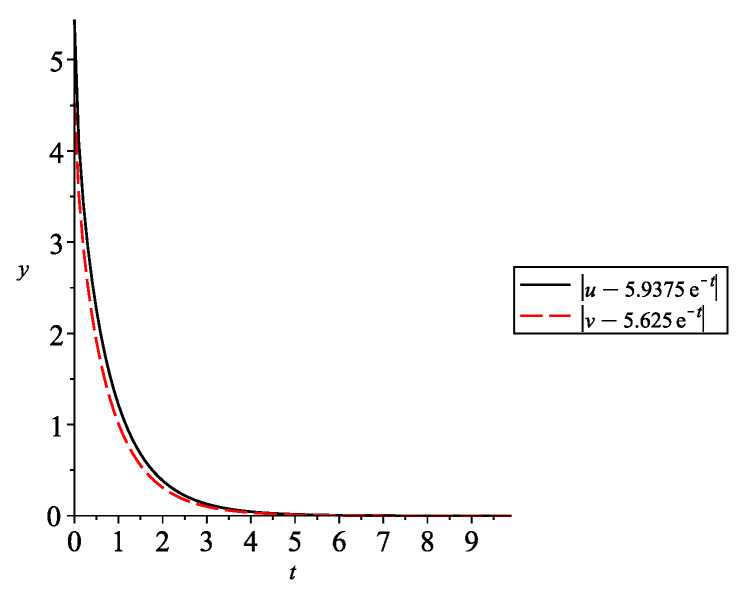
Convergence of the solution of (23) to the equilibrium $\left(5.9375e^{-t},5.625e^{-t}\right)$.

Model (23) is in the form of (9) with N=2, $h_{1}\left(s\right)=h_{2}\left(s\right)=s$, $d_{1}=\alpha ,\;d_{2}=\delta$, b=c=0. Thus, $\gamma_{1}=\gamma_{2}=1$, $\mu_{1}=\mu_{2}=1$ and

$$-2d_{k}+\sum_{j=1}^{N}\gamma_{j}\left|a_{kj}\right|+\frac{\mu_{N+k}}{\mu_{k}}c_{k}=-2\alpha +p<0,\;k=1,$$

and

$$-2d_{k}+\sum_{j=1}^{N}\gamma_{j}\left|a_{kj}\right|+\frac{\mu_{N+k}}{\mu_{k}}c_{k}=-2\delta +\beta GK<0,\;k=2,$$

i.e., assumptions (A1) and (A2) are satisfied if 0.5p<α and 0.5βGK<δ. In other words, the DNA repair rate δ and the expressome (proteome, metabolome) turnover rate, *c*, have to be large enough. In Figure 6, the graphs of the solution (u,v) are given with q=0.3,ρ=0.5, p=1,α=0.9,A=1,β=2,K=1,δ=0.8,G=0.5,B=2, and the initial values u(0)=0.5, v(0)=1.1. Then, the equilibrium is $\left(\tilde{u},\tilde{v}\right)=\left(\frac{1.9}{0.32}e^{-t},\frac{1.8}{0.32}e^{-t}\right))=\left(5.9375e^{-t},5.625e^{-t}\right).$

Note that the model (23) in the case of ordinary derivatives is studied in [2] with the more restrictive assumption KpβG<cδ.

## 6. Conclusions

A new gene-regulated model is set up. The dynamics is decribed by the generalized proportional Caputo fractional derivative. The equilibrium is defined in an appropriate way. In the general case, the classical definition of the equilibrium differs. The generalized exponential stability is introduced and studied via the application of Lyapunov functions and their generalized Caputo proportional fractional derivatives. In connection with the application of Lyapunov functions to fractional type models, some properties of absolute values Lyapunov functions and their fractional derivatives are discussed. Several examples are provided to illustrate the properties. The advantages of the application of the quadratic Lyapunov functions are considered, and sufficient conditions for generalized exponential stability are obtained. Some examples illustrate the theoretical results and the dependence of the fractional derivative on the behavior of the solutions.

## Data Availability

Not applicable.
